# Should I speak up? How trust in leaders and leader–leader exchanges influence nurses' voice behaviour

**DOI:** 10.1002/nop2.2101

**Published:** 2024-01-31

**Authors:** Kang‐I. Chao, Jen‐Wei Cheng, Hung‐Chieh Yen, Shih‐Hao Lu

**Affiliations:** ^1^ Department of Business Administration National Taiwan University of Science and Technology Taipei Taiwan; ^2^ Department of Counseling, Clinical and Industrial/Organizational Psychology Ming Chuan University Taoyuan Taiwan

**Keywords:** leader‐leader exchange, voice behaviour

## Abstract

**Aim:**

Discussing the nurses' voice behaviour could support the managers in making the right decisions and solve problems.

**Design:**

This was a discursive paper.

**Methods:**

The discursive was based on reviewing the literature.

**Results:**

Nurses play a critical role in offering useful constructive advice, which leads to management figuring out and solving problems immediately for the purpose of bettering the working environment. Therefore, we assert that trust in leadership and the leader–leader exchange system also plays a critical role in enforcing voice behaviour. Trust is a crucial aspect of voice behaviour, and integrated trust in leadership and leader–leader exchange as a possible practical suggestion for the fostering of voice behaviour are proposed. Nurse managers must maintain a sense of reciprocal moral obligation in order to nurture value‐driven voice behaviour. It is important that open dialogue, active listening and trust in leadership exist. Nurse managers must consider ways to foster mutual trust, and support and enable nurses to use voice behaviour in everyday practice.

## 1 INTRODUCTION

In every country, regardless of socio‐economic development, nursing is a front‐line defence against the spread of diseases and the alleviation of suffering during and after disease treatment (Buheji & Buhaid, 2020). In most hospitals, more than half the employees are nurses, and their jobs can include almost anything, from reporting a toilet clog in the ward to dealing with medical disputes and patients' health care (Schwerdtle et al., 2020; Ulrich et al., 2019). They are everywhere in the hospital and see everything in the hospital; therefore, if these nurses are willing to let their ‘voice’ be heard or are willing to help point out issues, many problems in a hospital may be solved a lot sooner or even easier (Jun et al., 2021).

The COVID‐19 pandemic has increased awareness of the important role that nurses play. The current paper is based on the assumption that nurses' voice behaviour supports the overall productivity of nurses during demanding periods, such as a pandemic. Thus, to enhance overall effectiveness in our responses to pandemics, we need to work on increasing nurses' voice capacity to help them to cope with the high levels of work pressure associated with such fierce circumstances.

Voice behaviour is an action that inspires nurses to give constructive ideas to ensure management figures out and solves problems immediately to better the workplace environment. By doing so, nurses can be trained to improve in a stable working environment and managers can operate sustainably. We argue that nurse voice behaviour manifests diversely, ranging in the times, depth and width of voice content. These manifestations are influenced by the maturity of the working relationships and differences in trust inclinations between leaders and subordinates (such as trust in leadership and leader–leader exchange). An elucidation of the roles played by these factors makes an incremental contribution to our overall understanding of nurses' voice behaviour.

The COVID‐19 pandemic has highlighted the importance of nursing care for the maintenance of life and the right to health, and the immediate voice behaviour is in line with the call for the appreciation of nursing professionals currently working at the forefront of the fight against COVID‐19 (Oliveira et al., 2020). It is necessary for nursing staff to recognize their own value, engage in ongoing voice behaviour, and be aware of environmental changes and their impacts. This study contributes to these goals by rethinking strategies for valuing nursing.

## 2 THEORETICAL OVERVIEW

The discussion in this article draws on social exchange theory and social information processing theory (Salancik & Pfeffer, 1978). Early studies emphasized the social exchange context of leader–member exchange (LMX). This then expanded to the cross‐level analysis of leader–leader exchange (LLX) (Chen & Lin, 2018). Thus, this current article is based on the idea that LMX, LLX and trust in leadership (TL) follow the norms of human relationships.

TL will affect a subordinate's evaluation of whether the gains or losses arising from cooperation with their immediate supervisor are within the permissible range (McKnight et al., 1998). Therefore, we assert that TL reduces uncertainty because subordinates believe in their immediate supervisor's goodwill towards them. We propose that LLX is more important, especially in voice behaviour, when leaders are trustworthy and when the social exchange context is uncertain because both LLX and TL involve an exchange of resources, particularly social capital. Thus, LLX and TL are complex phenomena occurring within a larger context of intricate social exchanges. We considered that the flow of social capital is therefore a decisive factor of voice behaviour.

**FIGURE 1:**
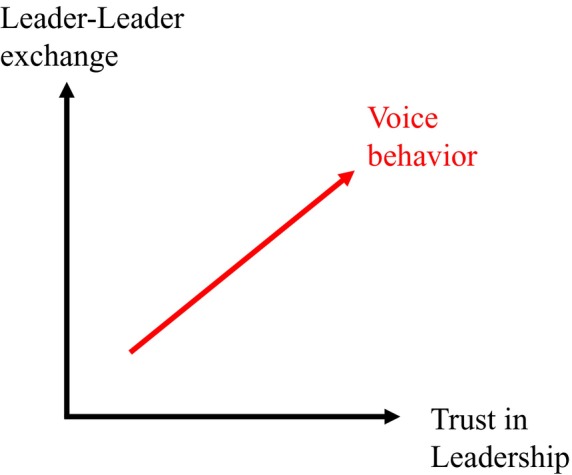
Influence of TL and LLX on voice behaviour.

Moreover, in cases of high‐quality LLX, the subordinate would perceive more utility and less risk in communicating directly with their immediate supervisor. Higher levels of trust lower the risk of exchanges between subordinates and immediate supervisors and thereby enhance cooperation. Subordinates would likely feel that such supervisors can help to resolve or act on subordinates' work‐related concerns and problems. Consequently, when both the quality of LLX and TL are high, employees are likely willing to express themselves openly to their immediate supervisor. That is, voice behaviour will be facilitated (Figure 1).

## 3 TRUST IN LEADERSHIP AND VOICE BEHAVIOUR

Hospital operation depends on the nurses' dedication and hard work (Cooper et al., 2021). By stimulating the nurses' enthusiasm, organizational effectiveness can be boosted (Zigarmi et al., 2009). Nurses' improvement of work performance not only relies on their professional skills, but also requires them to often observe the floundering problems at work and be willing to express constructive ideas (Lin & Johnson, 2015). In addition, the head nurse plays an important role and has the right to accept and implement the nurses' suggestions (Wei et al., 2019). In this context, nurses' expression of constructive, work‐related ideas to supervisors (Li et al., 2018), that is, voice, plays a critical role in linking nurses' own knowledge and insights with head nurses' influence (He et al., 2020). Furthermore, even though nurses might aim to use their voices in a positive manner to express the work‐related status quo, it can be associated with interpersonal risks as any voice behaviour of concern about current work practices might be seen as an implicit criticism of supervisors, and thus might lead to interpersonal rejection or even sanctions at work (Kim et al., 2019).

Morrison (2011) observed that there are two factors that may influence nurse's willingness to use their voice efficacy and safety. The efficacy of voice means nurses expect to be rewarded after voicing kindly, and the safety of voice means nurses consider whether the voice behaviour brings them risks, damages or benefits. Given these characteristics of voice, Tangirala and Ramanujam (2012) indicated that even when nurses are highly motivated in the workplace, they tend to speak up only when they perceive trust in their leader, and are thus likely to react to their ideas in a positive way and act to implement them (Kül & Sönmez, 2021). The quality of dyadic social exchange with the supervisor is a key determinant of subordinates' level of voice engagement, as well as their belief in the leader's positive intentions towards them (Lee et al., 2019). Therefore, trust in leadership and leader–member exchange perspectives form a natural starting point for an interpretation of employees' voice behaviours (Carnevale et al., 2020).

Trust always plays an important part in organizational management, especially in a hospital environment, because people are dealing with the life and death on a daily basis. Moreover, relevant research explained that nurses' trust in leadership, that is, the head nurse, can enhance the cooperation between them and lead to better outcomes (Afsar & Shahjehan, 2018). Head nurses have a key function in the organizational structure to communicate with superiors, and they have an influence on the nurses around them, give appropriate rewards to nurses and mobilize available resources in order to carry out work smoothly so that all stakeholders can engage with work and achieve the organizational goals (Emiralioğlu & Sönmez, 2021). The nurses' trust in leadership can reduce uncertainty because nurses believe in the head nurse's goodwill (Frahsa et al., 2021). On one hand, the nurse trusts the head nurse, which may increase their willingness to take a voice; on the other hand, before using their voice, the nurse has already considered the head nurse's trustworthiness, and whether the gains or losses arising from speaking up to the head nurse are within the permissible range (McKnight et al., 1998).

Therefore, nurses' trust in the head nurse can strengthen their relationship commitments. Trust between them can lower the risk of exchanges between nurses and head nurses and enhance cooperation. Since the head nurse's trustworthiness has made the nurses trust in their goodwill and reliability, she/he will have more confidence in their voice behaviour (Ng & Feldman, 2013), whilst this interpersonal relationship originates from the trust relationship between two parties. After they have established shared values and common goals, a high degree of trust can propel both parties to work for their mutual benefits at the expense of their own temporary advantages and cooperate to seek long‐term profits. Thus, the present commentary holds the first viewpoint that the key to nurses' proactive voice behaviour is that the degree of trust in the leader, particularly the head nurse, will be positively related to voice behaviour.

## 4 INTERACTION BETWEEN TRUST IN LEADERSHIP, LEADER–LEADER EXCHANGE AND VOICE BEHAVIOUR

In addition, most of the head nurses hold a great deal of responsibility because they are the life and soul of a ward. They have to deal with the relationships or even personal problems between different nurses, manage all the issues in the ward and execute the orders from higher management. Moreover, they have to solve all the problems relating to the ward. As a result, the status and influence of the head nurse in the organization are particularly important for the nurses (Huang et al., 2021). Therefore, we considered the relationship between trust in leadership and voice behaviour influenced by the leader's leader–leader exchange quality (Van Dyne et al., 2008). The present commentary holds a viewpoint that the key to nurses' proactive voice behaviour is not only the degree of trust in their leadership, but also in the nurses' examination of the head nurse's social capital of leader–leader exchange quality in the organization. These are the decisive factors for the interaction of voice behaviour between the nurses and head nurses.

Leader–leader exchange is a concept based on leader–member exchange, which indicates that the head nurse's social exchange relationship does not only exist between the head nurse and the nurse, but also between the head nurse and the superior leader (Zhou et al., 2012). The leader–leader exchange is a relationship of resource exchange between the head nurse and superior leader, and a pleasant relationship will affect the trust and the maintenance of good interactions between the head nurse and nurses at work. Although leader–leader exchange is similar to leader–member exchange, the difference lies in the power and responsibilities conferred by the organization. A high‐quality leader–leader exchange relationship will promote leader–member exchange and inspire nurses to come up with practical ideas (Botero & Van Dyne, 2009). Therefore, a high‐quality leader–leader exchange relationship represents that besides his/her own resources, the head nurse possesses extra beneficial resources gained from the social exchange relationship with the superior leader (Herdman et al., 2017). Based on these reasons, we indicated that leader–leader exchange and trust in leadership integration increase head nurse's resources and authorization from the superior leader, and influence trust and information sharing of the nurse. Perceived leader–leader exchange impacted the voice behaviour towards the head nurse, which makes nurses consider the effectiveness of speaking up to the head nurse. When leader–leader exchange was perceived to be high, as well as the nurse's trust in leadership, the nurse potentially had a greater reciprocity‐driven obligation to share information with the head nurse (Mo & Shi, 2018). The influence of the head nurse in the organization might have two effects on nurses. First, nurses would likely feel that the head nurses can help resolve or act on nurses' behalf to solve work‐related concerns and problems (Ali et al., 2021). Head nurses often gain resources and status in the organization because of their social exchanges with superior leaders, that is, leader–leader exchange, so head nurses with high leader–leader exchange usually have a greater ability to initiate change in the workplace in response to nurses' suggestions and concerns. Therefore, nurses might have inclination to take their voice to the head nurses, based on their high‐quality relationships with superior leaders.

However, researchers have noted that subordinates tend to remain silent when their leaders do not possess the requisite influence to act on their suggestions or input (Tangirala & Ramanujam, 2012). Herdman et al. (2017) pointed out that the nature of leader–leader exchange is the head nurses' upward exchange relationship with their own boss, which served as an important ‘linking pin’. This meant when the head nurse establishes a high‐quality leader–leader exchange relationship with the superior leader, the nurses would receive the shield from the head nurse and share the head nurse's good fortune. In contrast, if the head nurse failed to build up a high‐quality leader–leader exchange relationship with his/her superior leader, the nurses would also need to take up the misfortune of the head nurse. By contrast, when head nurses have low leader–leader exchange, nurses may feel that their voice is not likely to be instrumental in getting things done within the organization. Therefore, even when nurses perceive high trust with the head nurses, they might feel that it would be ineffective to bring up issues or concerns to head nurses and instead keep quiet to protect the direct leader suffer reproach.

Furthermore, we proposed that when nurses have a poor trust in leadership with the head nurse, combined with a strong relationship between the head nurse and superior leader (high leader–leader exchange), their voice behaviour towards the head nurse will veer towards management‐oriented. Impression management tactics are commonly categorized as being self‐focused (e.g., promoting one's skills and abilities in an effort to appear competent) or other‐focused (e.g., demonstrating values one has in common with another person to appear likable). Specifically, when nurses experience self‐protection concerns and believe that presenting their true selves in an interaction would not be in their best interest, they might be tempted to engage in such impression management behaviours. Employees may have to choose between multiple images of themselves or perhaps even a slightly exaggerated or false expression of themselves. This is especially true when nurses are concerned about the social exchange quality, which is at stake in both the leader–leader exchange and trust in leadership, where they may be more motivated to manage their impression. As such, it may be that self‐protection concerns faced by the social exchange (i.e., high leader–leader exchange and low trust in leadership) could actually be an underlying mechanism associated with nurses' choice to engage in voice behaviour through the impression management tactics.

## 5 CONCLUSION

The interaction of voice behaviour, trust in leadership and leader–leader exchange are seen as multidimensional, and applying the framework used in this paper to future research may deepen its interpretation and true value.

We propose that leaders' social capital, which is captured by higher quality LLX, is related to how well leaders can empower their teams and individual subordinates and therefore facilitate their subordinates' voice behaviour. Thus, organizations should help leaders to develop skills for building better and stronger relationships with their own superiors, which could relate to increases in the perceived support of their teams and their individual subordinates.

We also found that leaders' dyadic relationships with their subordinates are more likely to positively relate to subordinates' sense of trustworthiness when leaders also have positive relationships with their own superiors. This means organizations should aim to develop a broader climate that encourages trust and supportive relationships across organizational levels (Zhou et al., 2012). It is important to note the possibility that having a trustworthy team and trustworthy subordinates could improve the leader's image in the eyes of their superiors; thus, they are more likely to have a higher quality LLX with the upper level. As such, developing and maintaining a good relationship with one's subordinates might benefit the supervisors themselves as well.

On the other hand, it may provide guidance for voice behaviour in daily practice during a pandemic. Voice behaviour, willingness of efficacy and safety should be supplemented with trust in leadership and leader–leader exchange influence, that is, speaking up, management‐oriented impression and keeping silent.

## 6 IMPLICATIONS FOR NURSING MANAGEMENT

Head nurses who exhibit trustworthy behaviour and resilience in response to this pandemic can powerfully influence the actions of others. Nonetheless, the social exchange quality and dealing with the nurses' voice behaviour towards nurse managers in the aftermath of COVID‐19 will require renewed attention and proactive planning (Arnetz et al., 2020). Supervisors should therefore be aware of nurses' psychological stress whilst seeking to understand their cognition of different types of relationship scenarios to help them to manifest voice behaviour. Therefore, in order to face these challenges, it is crucial for nurse managers to foster trust in leadership, maintain the social exchange quality and exhibit professional resilience whilst inspiring, motivating and empowering their nursing team. The importance of having open dialogue and understanding the demands of nurses, whilst motivating voice behaviour and professional resilience must not be underestimated. Ultimately, nurse managers need to act as a moderator to identify the voice needs of staff, reduce scruple and support voice in a caring and initiative manner. However, for this to occur, nurse managers need to ensure support and voice behaviour are available to head nurses and nurses in the form of education, training, peer support and coaching. This is essential as we cannot support nurses without supporting head nurses, and the support for nurses can only be as good as the support of their superiors.

## CONFLICT OF INTEREST STATEMENT

None of the authors has any conflict of interest to declare.

## FUNDING INFORMATION

None.

## ETHICS STATEMENT

First, to the best of our knowledge, the named authors have no conflict of interest, financial or otherwise. Then, this material is the authors' own original work, which has not been previously published elsewhere. Furthermore, the paper is not currently being considered for publication elsewhere. Finally, the paper reflects the authors' own research and analysis in a truthful and complete manner.

## PATIENT CONSENT STATEMENT

As an analysis of the literature patient consent is not applicable to this article submission.

## Data Availability

Data sharing is not applicable to this article as no new data were created or analyzed in this study.
